# Sternal stress fracture presenting as acute chest pain

**DOI:** 10.1016/j.radcr.2023.09.029

**Published:** 2023-10-07

**Authors:** Chiew-Jen Ong, Talal Mourad, Parker Weiss, Ryan Martin, Grace Palaparty, Emad Allam

**Affiliations:** a Loyola University Chicago and Loyola University Medical Center, Department of Radiology, 2160 S 1st Ave, Maywood, IL, 60153, USA; b University of Illinois College of Medicine Peoria, Peoria, IL, 61605, USA; c Midwestern University Chicago College of Osteopathic Medicine, Downers Grove, IL, 60515, USA

**Keywords:** Sternal fracture, Stress fracture, Insufficiency fracture, Chest pain, Thoracic kyphosis, Compression fracture

## Abstract

Sternal fractures are rare and are typically caused by major trauma such as motor vehicle collisions. However, sternal insufficiency fractures can occur with minimal to no trauma in patients with exaggerated thoracic kyphosis from multiple thoracic compression fractures, especially in the setting of osteoporosis. We describe a case of a sternal insufficiency fracture that presented as chest pain resembling a myocardial infarction. As sternal insufficiency fractures may vary in clinical presentation, this case demonstrates that radiologists should carefully evaluate the sternum, especially when risk factors are present. Furthermore, awareness and identification of these fractures can prevent unnecessary cardiac workups.

## Background

Insufficiency fractures are a subset of stress fractures that occur when a bone, which may have decreased mineral content and/or elastic resistance, is subjected to normal force. These fractures commonly affect the lower thoracic vertebrae, lumbar vertebrae, sacrum, and proximal femur. Less frequently, they can also occur in the sternum [1]. The clinical presentation of sternal insufficiency fractures varies greatly, ranging from no pain to severe pain that can resemble a myocardial infarction [2]. Sternal insufficiency fractures are often present in the setting of osteoporosis with high degrees of thoracic kyphosis and multiple thoracic compression fractures [2].

Here we describe a case of sternal insufficiency fracture in an elderly female who presented with chest pain and no history of trauma. Such a presentation may easily warrant cardiac evaluation, and awareness of the variable presentation and subtle appearance of sternal insufficiency fractures is important so that it can be considered in the differential diagnoses.

## Case presentation

An 89-year-old female presented to the emergency department (ED) with chest pain and hypertension. The patient also reported subacute to chronic back pain. The chest pain was worse with palpation and when attempting to raise her arms above her head. She was given ibuprofen in the ED, which provided some pain relief. She had a history of hypertension, hyperlipidemia, colon cancer, abdominal aortic aneurysm, Chronic obstructive pulmonary disease (COPD), and osteoporosis. There was no recent trauma.

**Fig. 1:**
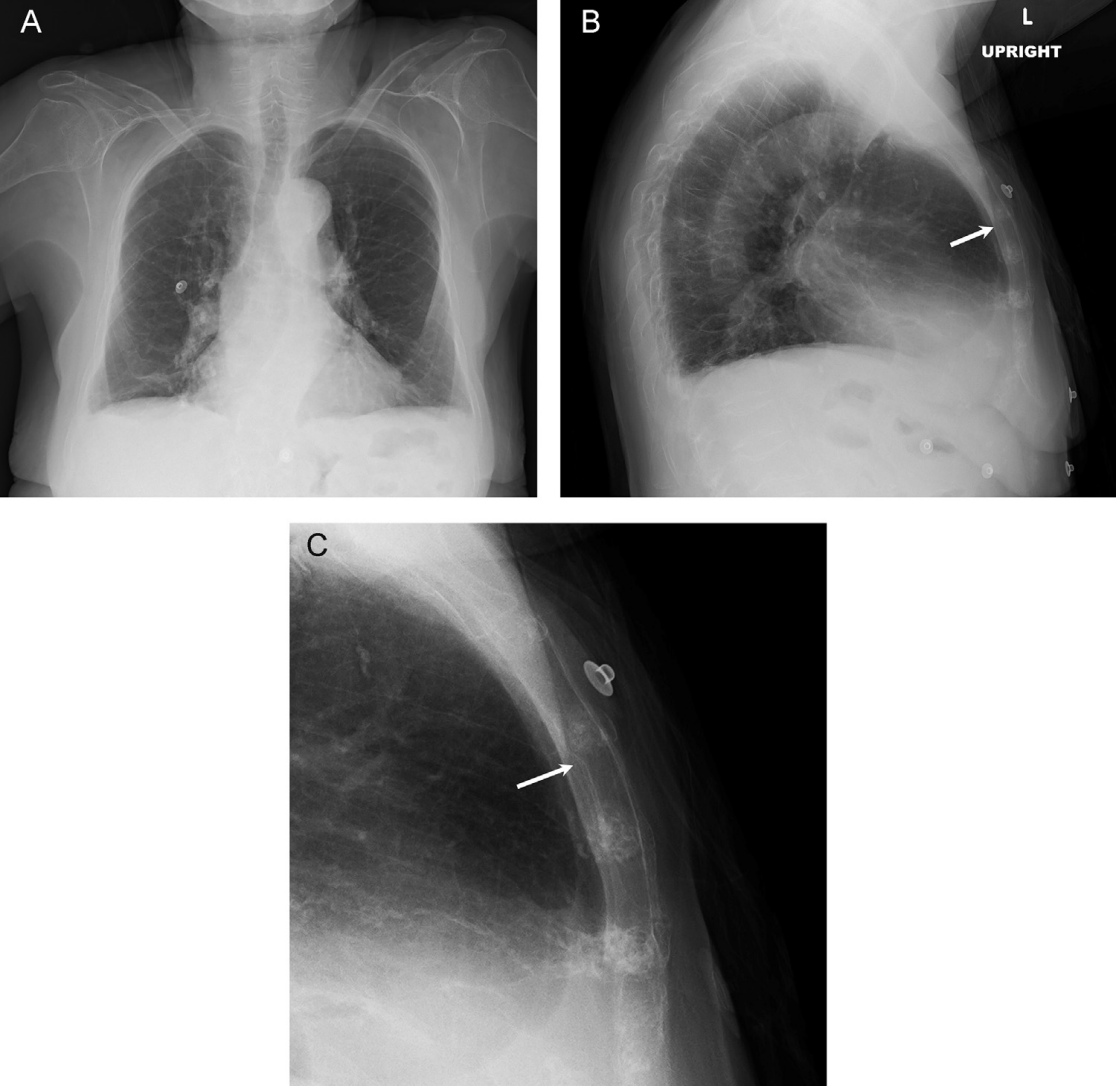
(A) PA radiograph of the chest shows no acute pulmonary abnormality. (B) Lateral radiograph of the chest also shows no acute pulmonary abnormality. Exaggerated thoracic kyphosis and multiple thoracic compression fractures are seen. There is subtle cortical offset at the posterior margin of the sternum (arrow). (C) Magnified view of lateral radiograph of the chest confirms cortical step-off at the posterior margin of the sternum (arrow). This is concerning for a nondisplaced sternal fracture.

**Fig. 2:**
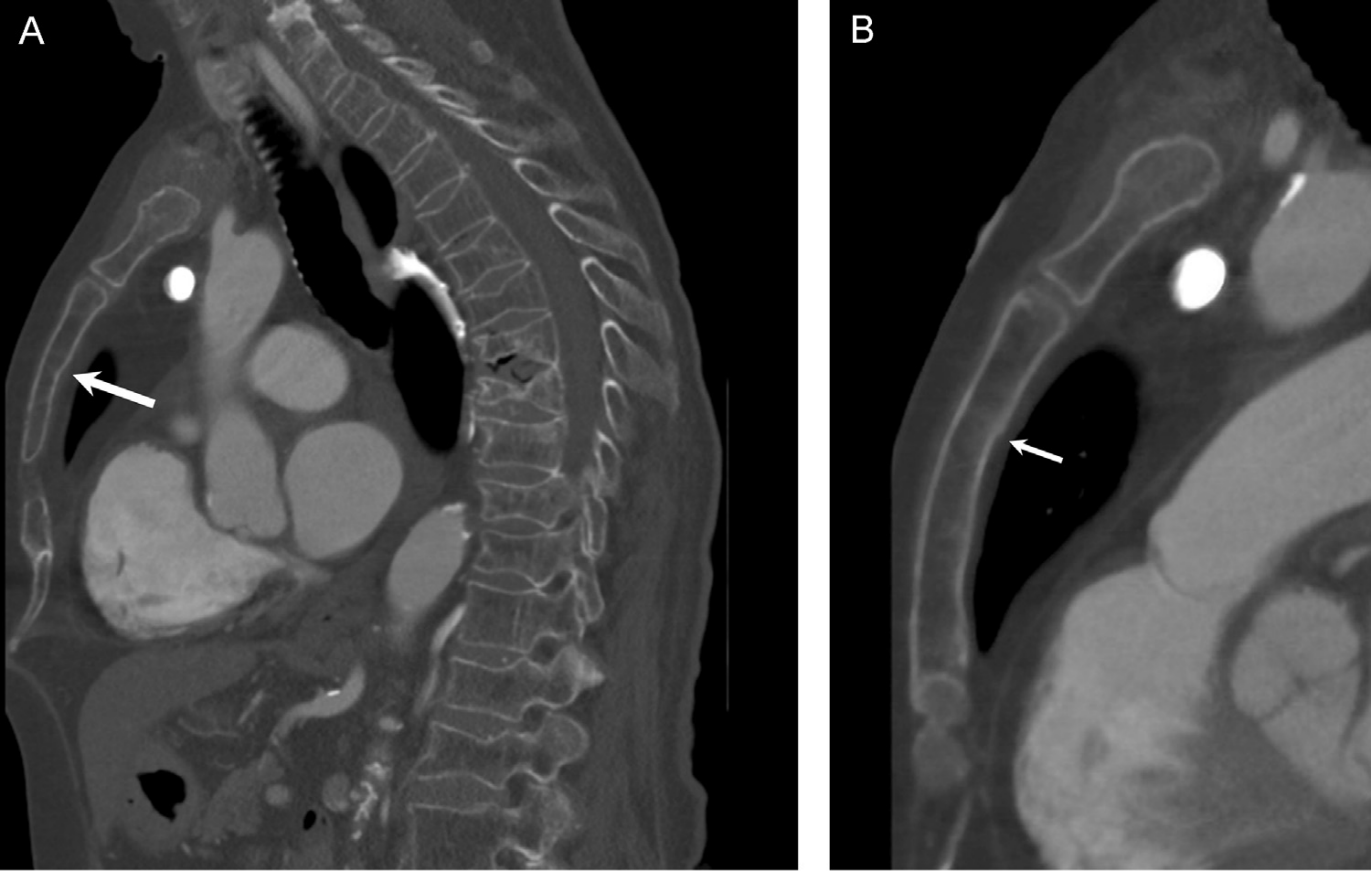
(A) Sagittal CT image of the chest with bone windows shows multiple thoracic compression fractures. These were chronic when compared to prior imaging. There is irregularity of the posterior margin of the sternum. (B) Sagittal CT image of the chest with attention to the sternum shows subtle cortical step-off at the posterior margin of the sternum, consistent with a nondisplaced fracture (arrow).

**Fig. 3:**
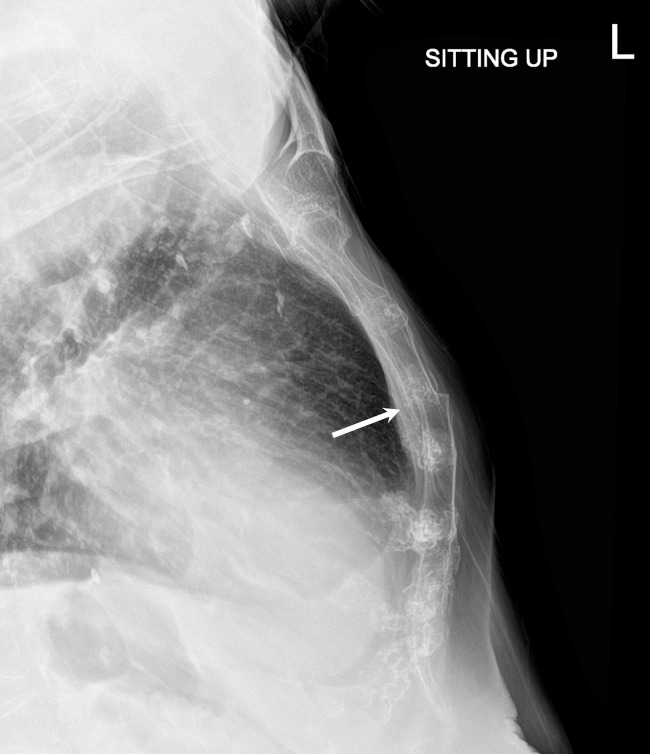
Follow-up lateral radiograph of the sternum shows increased displacement of the sternal fracture (arrow). This radiograph was obtained 2 weeks after the chest radiographs in Fig. 1.

In the ED, her troponin level was within normal limits and Electrocardiogram (EKG) showed no ST elevation myocardial infarction. Initial radiographs showed exaggerated thoracic kyphosis, multiple chronic thoracic compression deformities, and subtle sternal deformity (Fig. 1). CT angiogram of the chest performed on the same day revealed a nondisplaced sternal fracture (Fig. 2). She was started on conservative treatment with acetaminophen for pain management. Two weeks after the initial visit, her chest pain had improved but was still present. Follow-up sternal radiograph at the time showed a minimally displaced fracture of the sternal body (Fig. 3). The symptoms subsequently resolved with continued conservative treatment.

## Discussion

Sternal fractures are rare, accounting for only 0.5% of all fractures, and are typically caused by major trauma such as motor vehicle accidents [3]. Motor vehicle accidents are responsible for 90% of manubrium fractures [4]. Isolated sternal or manubrium fractures are even more rare [3]. Sternal insufficiency fractures may be challenging to diagnose due to their variable clinical presentations [2]. Less commonly, they may present as chest pain which can resemble a myocardial infarction [5], as was the case with this patient.

Sternal insufficiency fractures are often linked to high degrees of thoracic kyphosis from multiple thoracic compression fractures in the setting of severe osteoporosis [2]. The sternum and ribs act as the thoracic spine's fourth column of structural support, and an overriding, displaced sternal fracture could indicate a severe flexion-distraction injury to the spine with a high risk of deformity [6]. Osteoporosis-induced thoracic compression fractures often lead to thoracic kyphosis, which can increase the risk of sternal insufficiency fractures [7,8]. These fractures develop when osteopenic or osteoporotic bones are unable to withstand the stresses of daily activity, such as sudden forward angulation of the thoracic spine [7]. The stresses are then transmitted to the sternum by the ribs and clavicles, resulting in acute flexion-compression stress that may increase sternal fracture risk [3,^9^,10]. The sternum's flat bone structure, comprising two thin layers of compact bone surrounding trabecular bone, further makes it susceptible to such microarchitectural disruption [2]. Corticosteroid therapy and restricted mobility in COPD patients can also accelerate the loss of trabecular bone, resulting in progressive thoracic kyphosis and deforming stress to the sternum [11,12]. Coughing may also exacerbate this effect [11]. Furthermore, the fracture cascade phenomenon of osteoporosis may increase the risk of subsequent fractures [2,13].

It is important to recognize sternal fractures as a differential diagnosis for osteoporotic patients with chest pain. Clinical presentation can range from asymptomatic to severe pain [2]. In most cases, radiographs or CT imaging are used to diagnose sternal fractures [3]. However, studies have shown that radiographs alone may not be sufficient and could underestimate the extent of injury. When clinical suspicion is high, a negative chest radiograph should be followed by a CT and, when possible, 3-D CT scan reconstruction, which has been shown to be more sensitive and specific in detecting sternal fractures compared to both radiographs and 2-D CT scans [14], [15], [16].

Patients with uncomplicated sternal fractures may be discharged with oral pain medication [16,17], and if the pain persists, a periosteal catheter can be used to deliver continuous local anesthetics and/or opioids [18]. Surgical fixation may be required if the sternal fracture is complex or has overriding fragments [19,20]. Emergent surgery may be required if there is significant displacement of fracture fragments into the mediastinum with vascular compression/injury. If the chest pain and disability do not improve despite treatment, follow-up CT should be performed to assess for nonunion or pseudoarthrosis of the fracture [3].

Other complications associated with isolated sternal fractures include acute respiratory distress syndrome (ARDS), cardiac arrest, and pulmonary embolism [21]. Potential infectious complications associated with sternal fractures are mediastinitis and osteomyelitis, particularly in patients with a history of intravenous drug abuse [22,23]. However, the prognosis for isolated sternal fractures is typically favorable [24].

## Conclusion

Sternal insufficiency fractures are rare and are often missed partly due to inadequate visualization on plain radiographs as well as decreased awareness. The existing literature shows that sternal insufficiency fractures should be considered when chest pain is present in the setting of osteoporosis or multiple spinal compression fractures, as these are well-established risk factors. As the initial radiograph may be negative, our case demonstrates the value of using additional imaging modalities for diagnosis. Furthermore, our case demonstrates the association of sternal insufficiency fractures with osteoporosis-induced thoracic compression fractures, thoracic hyperkyphosis, as well as COPD. In conclusion, it is necessary for radiologists and clinicians to be cognizant of these fractures, especially in the setting of one or more risk factors, and the diagnosis may require multiple imaging modalities.

## Patient consent

Informed consent for this case was obtained from the patient.
